# Effect of Rosemary Essential Oil and *Trichoderma koningiopsis* T-403 VOCs on Pathogenic Fungi Responsible for Ginseng Root Rot Disease

**DOI:** 10.4014/jmb.2002.02013

**Published:** 2020-04-09

**Authors:** Khalid Abdallah Hussein, Young-Don Lee, Jin Ho Joo

**Affiliations:** 1 Soil Biochemistry Lab, Department of Biological Environment, Kangwon National University, Gangwon-Do 24341, Republic of Korea; 2 Botany and Microbiology Department, Faculty of Science, Assiut University, 71516, Assiut, Egypt

**Keywords:** Rosemary, essential oil, VOCs, antifungal activity, ginseng root rot

## Abstract

Rosemary essential oil was evaluated for antifungal potentiality against six major ginseng pathogens: *Sclerotinia sclerotiorum, Sclerotinia nivalis*, *Cylindrocarpon destructans*, *Alternaria panax*, *Botrytis cinerea*, and *Fusarium oxysporum*. The in vitro fungicidal effects of two commonly used fungicides, namely mancozeb and fenhexamid, and the volatile organic compounds (VOCs) of *Trichoderma koningiopsis* T-403 on the mycelial growth were investigated. The results showed that rosemary essential oil is active against all of the pathogenic strains of ginseng root rot, whereas rosemary oil displayed high ability to inhibit the *Sclerotinia* spp. growth. The highest sensitivity was *S. nivalis*, with complete inhibition of growth at 0.1% v/v of rosemary oil, followed by *Alternaria panax,* which exhibited 100% inhibition at 0.3% v/v of the oil. Minimum inhibitory concentrations (MICs) of rosemary oil ranged from 0.1 % to 0.5 % (v/v). Chemical analysis using GC-MS showed the presence of thirty-two constituents within rosemary oil from *R. officinals* L. Camphore type is the most frequent sesquiterpene in rosemary oil composition. Mancozeb and fenhexamid showed their highest inhibition effect (45% and 30%, respectively) against *A. panax*. *T. koningiopsis* T-403 showed its highest inhibition effect (84%) against *C. destructans* isolate. This study may expedite the application of antifungal natural substances from rosemary and *Trichoderma* in the prevention and control of phytopathogenic strains in ginseng root infections.

## Introduction

Korean ginseng (*Panax ginseng* L.) is mainly cultivated in South Korea, China, and the US, and currently distributed to many countries around the world. It is a very important perennial herb and has been widely used as a medicine and dietary supplement in Asia for more than a thousand years [1]. Ginseng plants are vulnerable to various soil-borne diseases, primarily several species of fungal pathogens that can cause root rot disease [1,2]. Opportunistic fungal pathogens usually attack the weakened plant parts and ginseng plant seedlings are therefore vulnerable to *Alternaria* and *Fusarium* infections [2]. Root rot fungi are the most potentially destructive as they can attack and ruin the roots of older plants. Several species of fungi, such as *Alternaria*, *Botrytis*, *Cylindrocarpon*, *Fusarium*, and *Sclerotinia,* have been involved in some forms of ginseng root rot [2]. Therefore, to overcome these problems, most ginseng fields are treated with synthetic agricultural chemicals [3, 4]. However, there is strong disagreement regarding the safety aspects of these synthetic chemicals since they are considered as carcinogenic and responsible for many diseases as well as residual toxicity [5]. Furthermore, the indiscriminate use of these synthetic chemicals has led to the development of multiple resistances. Recently, interest has been growing with regard to phytochemicals as a new source of natural antimicrobial agents.

Essential oils are naturally aromatic and volatile complex mixtures obtained from plant materials [6]. Most all essential oils are classified as Generally Recognized as Safe (GRAS) and possess low risk of resistance evolution in pathogenic microorganisms [7]. Essential oils are known as broad-spectrum antimicrobials, i.e., they are effective against multiple species of pathogenic fungi [8-10]. This is possible because one essential oil can possess multiple active ingredients with antimicrobial properties [11]. In general, essential oils possess greater antifungal activity because of the synergistic effect with their active components; therefore, they are more promising for commercial applications than single compounds [12]. *Rosmarinus officinalis* L. (family, Lamiaceae), widely known as rosemary, is one of the most popular culinary plants cultivated all over the world [13]. Rosemary is widely recognized as one of the spices having a high number of active ingredients [5]. The principal constituents of rosemary essential oil possess pharmacological value in addition to having antioxidant [13,14] and antimicrobial properties [15-18].

Volatile-mediated interactions between microbes and plants have been gaining more attention in agriculture [19]. For example, Campos *et al*. [20] suggested the volatiles produced by interacting microorganisms as biological control agents for the control of plant pathogens. Furthermore, it is known that bacterial volatiles promote the growth of the plant and activate induced resistance against phytopathogens [21]. However, the effects of volatile organic compounds emitted by *Trichoderma* species on plant growth have been recognized only recently [22]. In this study, the in vitro fungicidal effects of rosemary essential oil, the volatile organic compounds (VOCs) of *T. koningiopsis* T-403, and two commonly used fungicides, namely mancozeb and fenhexamid, on mycelial growth of six soil-borne ginseng pathogenic fungi, were investigated.

## Materials and Methods

### Sampling and Fungal Cultures

The fungi used in this investigation are pathogenic strains collected from the infected roots of Korean ginseng (*Panax ginseng*) in a soil biochemistry lab at Kangwon National University, Korea. The ginseng plants were collected from many different Korean ginseng fields. Plants were pulled up, put into zipper bags, and conveyed to the laboratory. Growth of isolates was maintained on potato dextrose agar (PDA), with incubation at 25°C for 7 days. The isolated fungal strains from the infected roots of *P. ginseng* were identified by direct microscopic examination and the features of the culture according to Domsch *et al*. [23] and Moubasher [24]. Six phytopathogenic fungi were chosen for this study, *i.e*., *Sclerotinia sclerotiorum, S. nivalis*, *C. destructans*, *A. panax*, *B. cinerea*, and *F. oxysporum*, and the samples were indentified and confirmed as pathogenic strains by the Korean Agricultural Cultural Collection (KACC), Jeonju, Korea. *T. koningiopsis* T-403 strain, used for its VOC activity, was isolated from ginseng rhizosphere soil and was selected based on its plant growth-promoting traits (unpublished data).

### Pathogen Rosemary Oil-Exposure Bioassay

The test was conducted using 100% pure essential oil of rosemary (*Rosmarinus officinalis*, family: Lamiaceae) purchased from a producer (Sydney Essential Oil Co. Ltd., Australia). For this technique, different dilutions of essential oil were prepared in PDA solution and then a mycelial disc of the pathogen was inoculated on the PDA using a borer with 5 mm diameter, as illustrated by Jiang [25]. Antifungal tests were conducted in triplicate and average values were used. Agar dilution is a suitable method to produce a saturated moisture atmosphere and adjust volatility [26]. PDA plates without essential oil were used as a control. The cultures were kept at 25°C for 10 days. Observations on the antifungal activities of essential oil on the pathogenic fungi were reported after 10 days and the inhibition indices were calculated according to the formula of Messgo-Moumene *et al*. [27]:


$${\text{Inhibition percentage (\%) = C}}_{1}{\text{-C}}_{2}{\text{/C}}_{1}\;\times \text{100}$$

where, C_1_ is the colony area of an uninhibited phytopathogenic fungus in the control, and C_2_ is the colony area of a phytopathogenic fungus in the dual culture.

### Pathogen-Trichoderma VOC-Exposure Bioassay

The *Trichoderma* isolate was inoculated in malt extract agar (MEA) medium and incubated at 25°C. One day later, the lid of the Petri dish was exchanged with the bottom of a 3-day-old PDA culture of the phytopathogenic fungi. The halves of the two cultures were sealed together using parafilm tape and incubated for 5 days. The linear growth of the test phytopathogenic fungi was measured. The bioassay was conducted in triplicate. The controls were only inoculated with the phytopathogenic fungi. All of the bioassay procedures were carried out under light- limited conditions to regulate the sporulation of *Trichoderma* [28]. The inhibition percentage was calculated relative to the control as mentioned above.

### Pathogen-Fungicide Assay

The inhibitory effects of mancozeb (75%, Indofil) and fenhexamid (50%, Indofil) on the fungal growth of pathogenic strains were investigated using agar-dilution method. Fungicide solutions were prepared in sterile deionized water and added to cool autoclaved PDA (approximately 50°C) to a final concentration of 20 μg ml^-1^ for each chemical fungicide. The mixtures were decanted into Petri dishes (100 mm × 15 mm) before hardening. A mycelial agar disc (5 mm in diameter) was cut off from the margin of the freshly growing mycelia of ginseng root- rot fungi and inoculated on the fungicide-amended PDA surface. The pathogen-fungicide assays were carried out in triplicate, and all cultures were incubated at 25°C for 10 days in darkness. Colony diameters were measured and the inhibition indices were calculated in comparison to the control (fungicide-free cultures) [27].

### GC-MS Analysis and Conditions

The analysis of rosemary oil was carried out on an Agilent GC-MS (7890A GC and GEOL JP/JMS-Q1050GC MSD) prepared with a splitless injection and a capillary DB-WAX MS column (30 × 0.32mm, 0.5 μm film thickness). The injection port temperature was maintained at 250°C and the column oven temperature of the program was set in a range of 50°C to 250°C (6 /°C min), then raised to 300°C (5°C /min), terminating with a 5 min isothermal at 300°C. Helium (1 ml/min) was the carrier gas and the mass spectrum was detected at 70 eV. The chemical composition was identified by comparison of the mass division patterns of the constituents to those of the WILEY reference standard data and libraries of NIST.

### Statistical Analysis

The obtained results were statistically analyzed by using SAS software [29] for all data using version 11.0 of Tukey’s test to compare the averages (*p* > 0.05).

## Results

### Antifungal Properties of Rosemary Oil

**Fig. 1:**
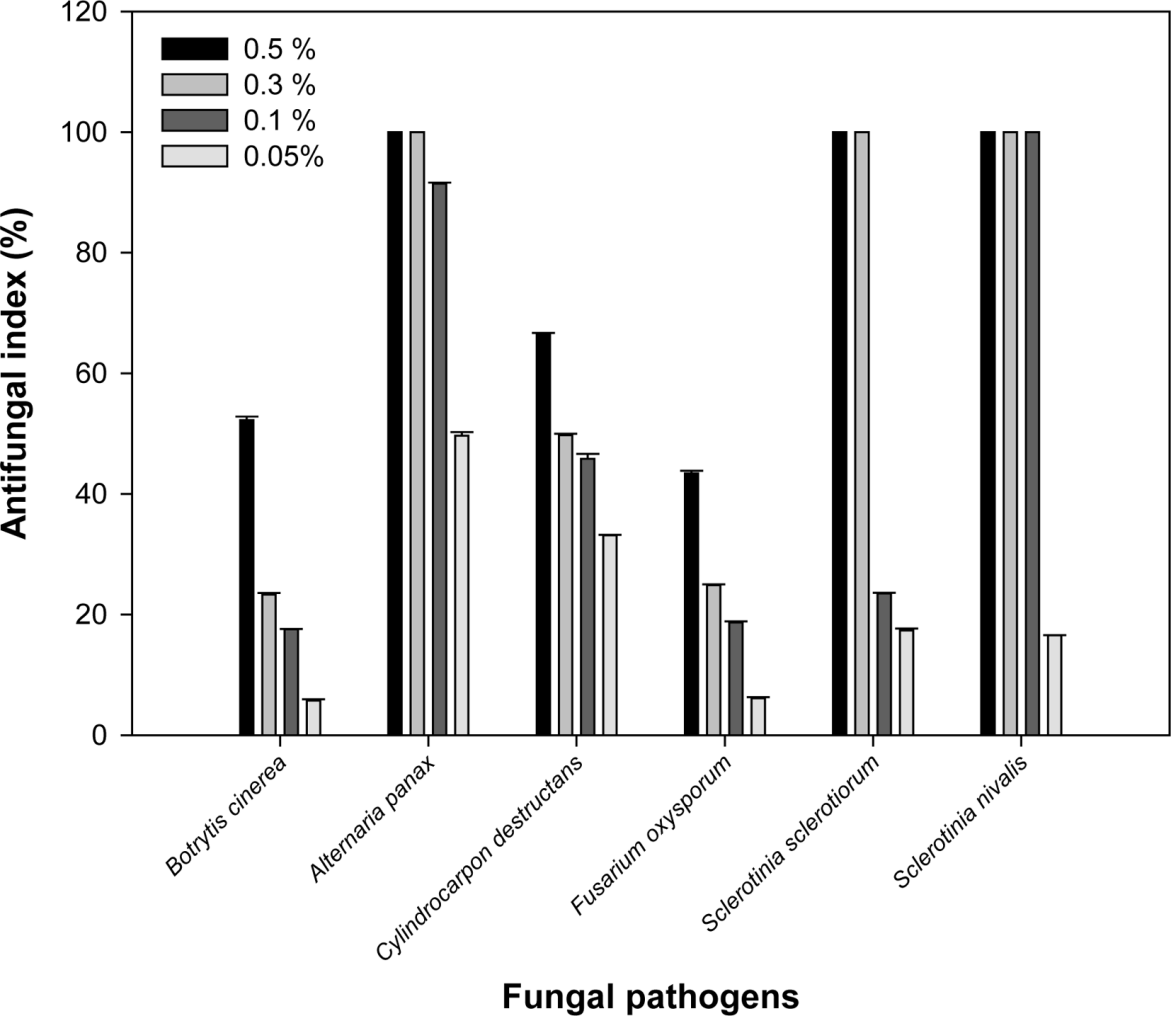
Antifungal activity of the essential oil of rosemary against ginseng root rot fungi. *B. cinerea*; *A. panax*; *C. destructans*; *F. oxysporum*; *S. sclerotiorum*; *S. nivalis*. The phytopathogenic fungi (agar dilution tests) were examined after 10 days and the inhibition percentages were calculated.

The essential oil exhibited significant antifungal properties against the tested pathogenic fungi. Rosemary oil was effective against *S. sclerotiorum, S. nivalis*, *A. panax*, and *C. destructans*. The highest antimicrobial activity was noticed against *S. nivalis* strain (100% inhibition) and the lowest antimicrobial effect was detected in *F. oxysporum* strain (43.4% inhibition) at 0.5% v/v of the oil. Rosemary oil at very low concentration (0.05% (v/v)) could inhibit the mycelial growth of *C. destructans* by inhibition percentage of (33%) and *A. panax* by inhibition percentage of 50%. Rosemary oil at 0.1% (v/v) showed complete inhibition only against the phytopathogen *S. nivalis*, and 91.5% inhibition to *A. panax*. The oil at 0.3% (v/v) revealed complete inhibition against *A. panax*, *S. sclerotiorum,* and *S. nivalis*, and only 25% inhibition to *F. oxysporum*, and 23% to *B. cinerea*. Rosemary oil at 0.3% (v/v) revealed 100% inhibition of mycelium growth to the ginseng pathogenic fungi *S. sclerotiorum,* and *S. nivalis* and 50% inhibition to *C. destructans*. Rosemary oil at 0.5% (v/v) showed 52% inhibition indices to *B. cinerea*, 66% to *C. destructans*, and 43% to *F. oxysporum*, and complete inhibition to *A. panax*, *S. sclerotiorum,* and *S. nivalis* (Fig. 1).

**Fig. 2:**
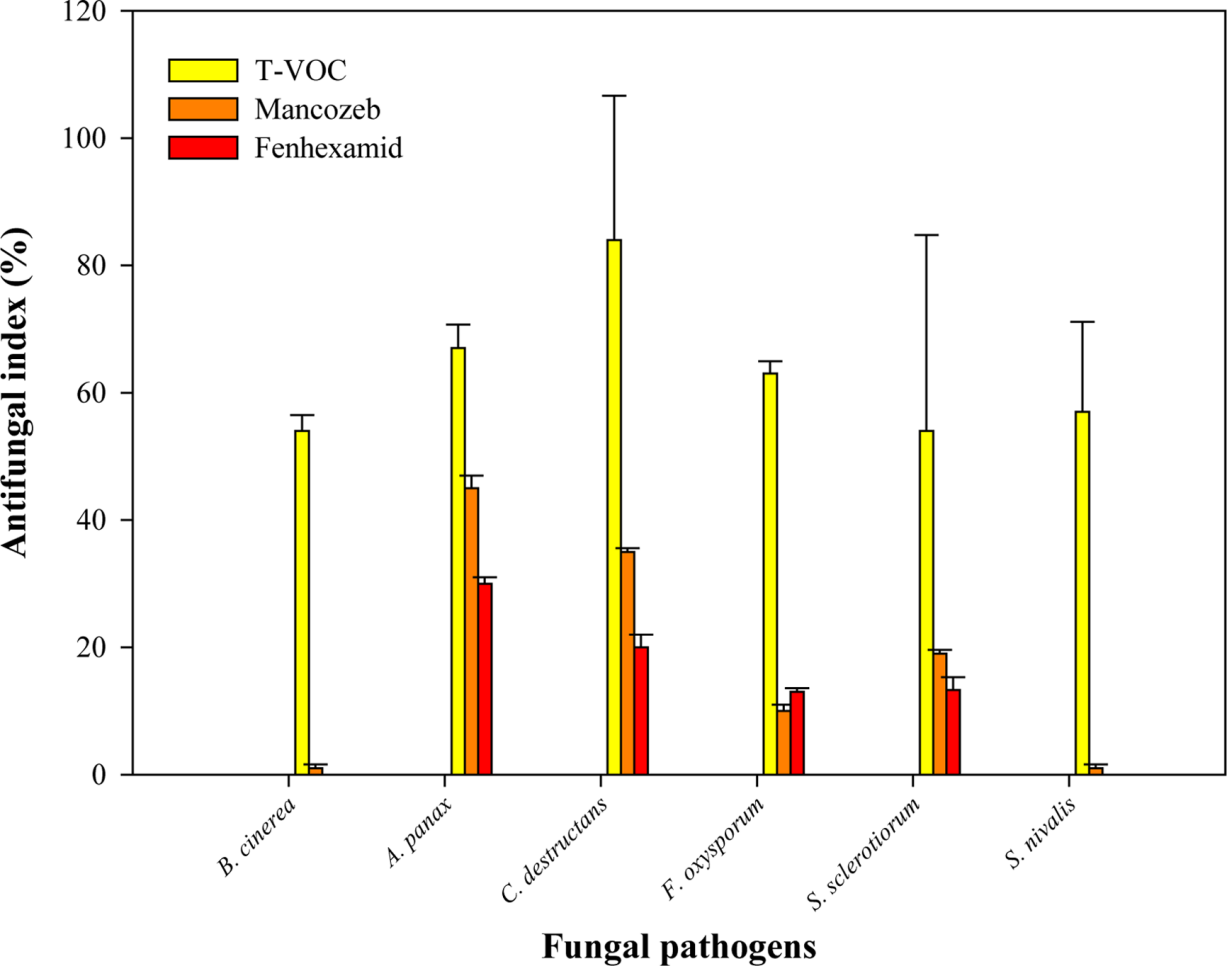
Antifungal activity of *T. koningiopsis* T-403 VOCs against ginseng-root rot fungi in comparison to fungicides (20 μg ml^-1^) on *B. cinerea*, *A. panax*, *C. destructans*, *F. oxysporum*, *S. sclerotiorum*, and *S. nivalis*. The antifungal indices were detected after 10 days.

Both fungicides mancozeb and fenhexamid showed their highest inhibition effect against *A. panax* (45% and 30%, respectively). However, their lowest inhibition effect was against *B. cinerea* (1% and 0%, respectively). *T. koningiopsis* T-403 VOCs showed their lowest inhibition effect also against *B. cinerea* (54.3%). Mancozeb was found to be the most active fungicide, inhibiting the growth of all studied isolates, showing 1% to 45.2% inhibition indices at a concentration of 20 μg ml^-1^. However, fenhexamid was the inferior fungicide, showing inhibition indices between 0% to 30% at the same concentration (Fig. 2). The chemical fungicides used in this study exhibited inhibition indices of less than 50% in mycelial growth at a concentration of 20 μg ml^-1^. However, the inhibition index of rosemary oil on the fungal growth was 100% at only 0.1% concentration in *S. nivalis*, while the *T. koningiopsis* T-403 VOCs showed inhibition range of 54.7% to 84% (Table 1).

### GC-MS Analysis

The relative components of main compositions in rosemary oil were a-pinene (12.9%), Bicyclo [2.2.1] heptane- 2-one, trimethyl (12.33%), Eucalyptol (11.21%), Camphene (11.04%), b-Pinene (8.09%), D-Limonene (6.16%), a-Phellandrene (1.01%), and β-Caryophyllene (0.38%) (Table 2).

## Discussion

Ginseng root rot can be caused by several different fungi; some of these fungi are uncommon and can cause more than one disease type and attack the seeds as well. At least twelve pathogenic fungal organisms attack *Panax ginseng* roots, *e.g*. *Botrytis*, *Fusarium*, *Cylindrocarpon*, and *Sclerotinia* [30]. In South Korea, limited studies have been focused on fungicide efficiency against phytopathogenic fungi [31]. The spectrum of the new antifungal agent is generally recognized by specific biological tests on various fungi allowing distinguishing tolerant and sensitive species. The natural polymorphism and accidental mutations may comprise an undeniable level of tolerance to some strains [32]. Mancozeb is a wide-range fungicide for many fungal pathogens, acting on several metabolic pathways in fungal cells [33]. It is a common multi-site dithiocarbamate fungicide, mostly used for preventing spore germination and susceptible to being washed away by rain or irrigation [33]. Fenhexamid is of the hydroxyanilide family [N-(2,3-dichloro-4-hydroxylphenyl)- 1-methylcyclohexanecarboxamide] that prevents RNA and DNA biosynthesis and cell division in fungi [32]. Our results showed that the highest inhibition effect of 20 μg ml^-1^ mancozeb was 45% against *A. panax*, while 20 μg ml^-1^ fenhexamid showed only 30% inhibition activity for the same fungus. Jose *et al*. [34] investigated the sensitivity of *venturia inaequalis* isolates to mancozeb in vitro and found that a concentration of only 0.6 μg ml^-1^ is proposed as a discriminatory dose for determination of sensitivity to mancozeb. Buck [35] used a combination of fungicides with phylloplane yeasts to improve the control of *B. cinerea* on geranium seedlings, and found that 7.5 μg ml^-1^ mancozeb did not improve the yeast biocontrol efficacy, and thiophanate-methyl fungicide had an adverse effect on the yeast at the same concentration. Hwang *et al*. [34] suggested only 10 μg ml^-1^ for the reduction of mancozeb residue in the postharvest treatments of fresh apples. Shin *et al*. [31] studied the inhibition indices of six fungicides at different concentrations (0.01, 0.1, 1, and 10 μg ml^-1^) on the mycelial growth of several *C. destructans* strains in vitro. Among the fungicides assessed was the dithiocarbamate fungicide mancozveb, which was highly effective on the conidial production and temperately effective on the hyphal growth of *C. destructans* [31].

**Fig. 3:**
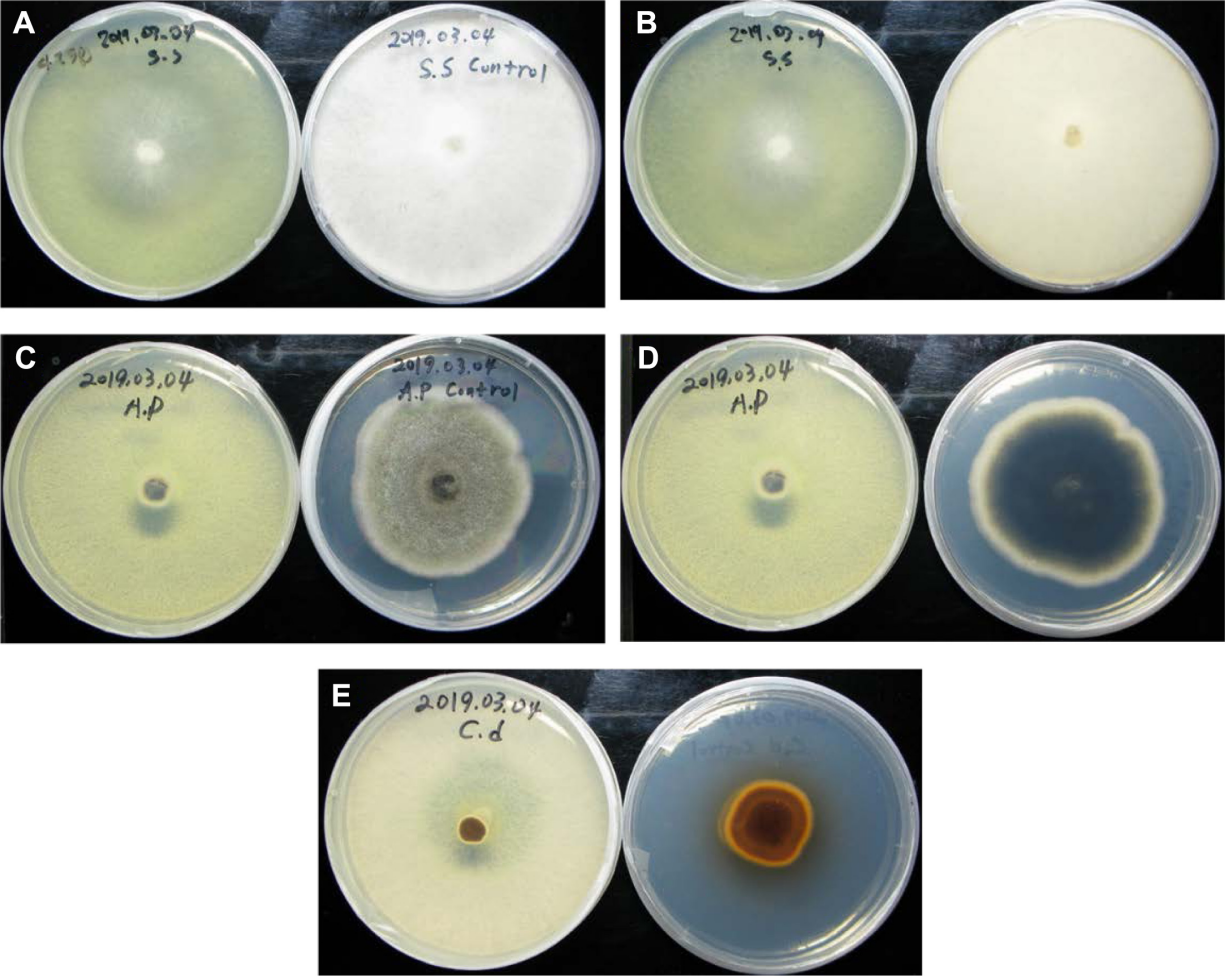
Pathogens-*T. koningiopsis* T-403 VOC-exposure bioassay in 10-days old cultures sealed with parafilm; A) *S. sclerotiorum* alone (right) and with *T. koningiopsis* T-403 (left)*;* B) *S. Sclerotiorum* (control reverse); C) *A. panax*; D) *A. panax* (control reverse); E) *C. destructans* (control reverse).

Walker *et al.* [34] estimated a new indigenous species causing gray mildew in the French vineyards with *B. cinerea* named *B. pseudocinerea,* which was originally resistant to fenhexamid. Recently, four different fenhexamid-resistant groups could be detected among the grey mold population [32]. Many *Trichoderma* species possess the ability to inhibit plant diseases and enhance plant growth and productivity by using an arsenal of mechanisms including myco-parasitism [35], antibiosis [36], induced systemic resistance [37], and supported nutrient efficiency [38]. However, only a few studies have concentrated on *Trichoderma* volatile compounds and their influence in terms of plant protection. In this study, *T. koningiopsis* T-403 VOCs showed high inhibition indices (84%) against *C. destructans* isolate (Fig. 3). *T. koningiopsis* T-403 was previously demonstrated by our laboratory group to have effective plant growth-promoting activity and inhibitory effect on the plant pathogens after screening many strains. VOCs released by *T. pseudokoningii* were correlated with the highest *Arabidopsis* growth promotion [39]. The volatile compounds emitted by *Trichoderma* can induce plant resistance to pathogens, thus improving plant health [39].

**Fig. 4:**
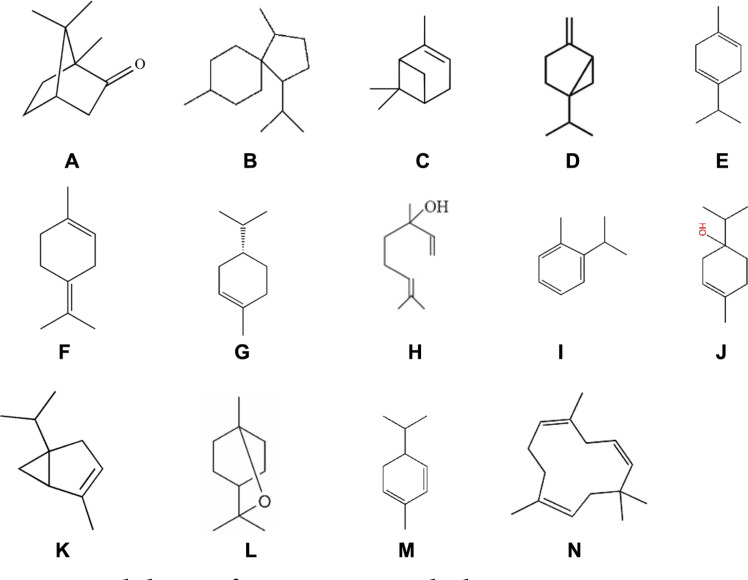
Main sesquiterpenes skeletons of rosemary essential oil. (**A**) camphore type, (**B**) acorane type, (**C**) pinene type, (**D**) sabinene type, (**E**) terpinene type, (**F**) terpinolene type, (**G**) limonene type, (**H**) linalool type, (**I**) cymene type, (**J**) terpineol type, (**K**) thujene type, (**L**) eucalyptol type, (**M**) phellandrene type, (**N**) caryophyllene type .

**Fig. 5:**
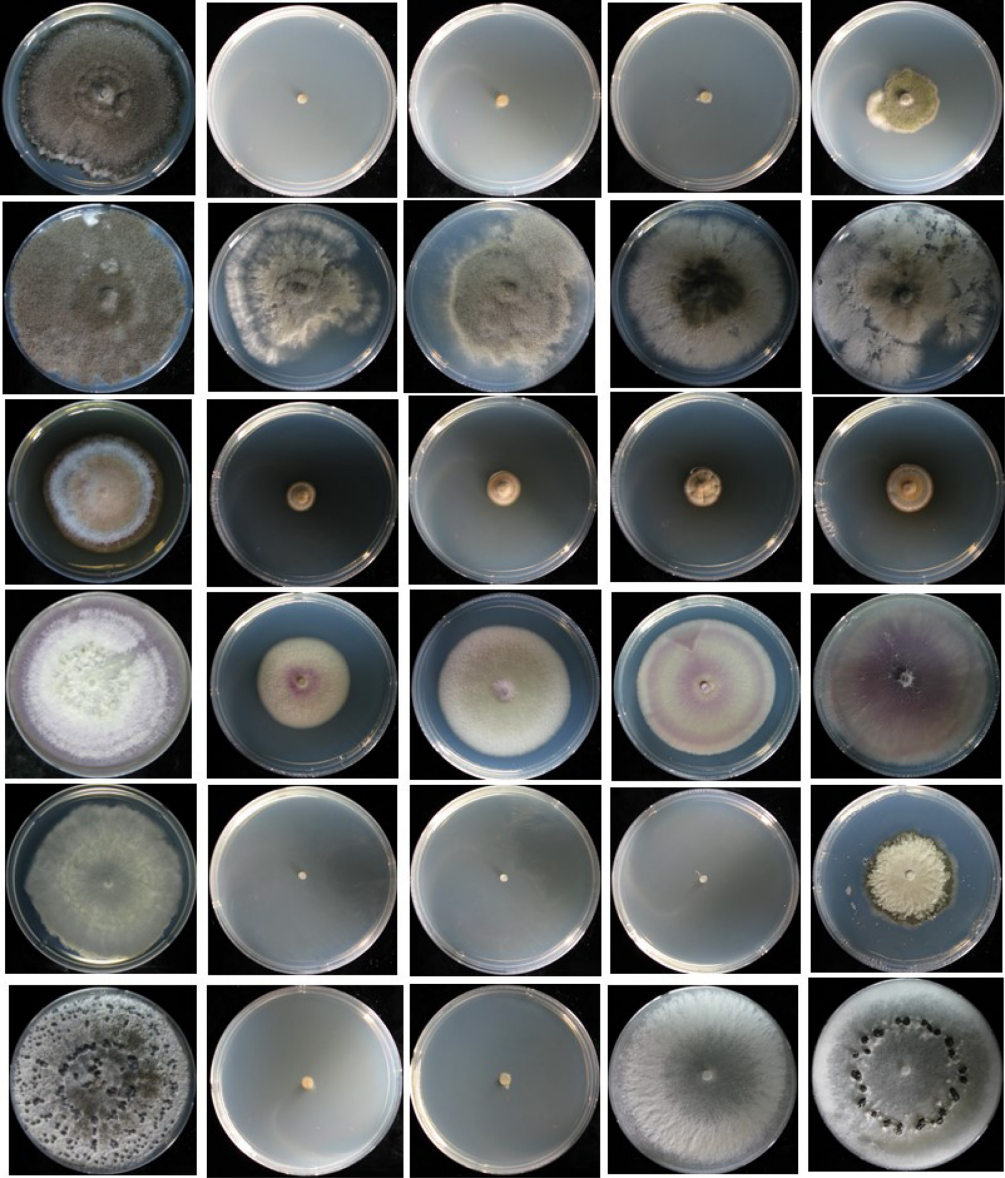
Antifungal activity of the rosemarys’ essential oil (left to right) Control, 0.5, 0.3, 0.1, and 0.05 % against ginseng root rot fungi (up to down) *B. cinerea*; *A. panax*; *C. destructans*; *F. oxysporum*; *S. nivalis; S. sclerotiorum*, on agar dilution test, phytopathogenic fungi were examined after 10 days and the inhibition percentage was calculated according to the formula of Messgo-Moumene *et al.* (2015).

Minimum inhibitory concentrations (MICs) of the essential oil from rosemary (*R. officinalis* L.) against six ginseng root rot fungi were determined. Rosemary oil possesses significant antifungal effects against all tested pathogenic fungi. Rosemary oil was potent against the fungi *A. panax*, *S. nivalis*, *S. sclerotiorum*, and *C. destructans*. The best antimicrobial activity was obtained for *S. nivalis* strain and the lowest effect was found in *F. oxysporum* strain (Table 1, Fig. 1). The dissimilar result of the essential oil effect against different fungal species may be due to their influence on organelles and biosynthesis rather than only a cell wall. Some previous studies have demonstrated that natural and synthetic antimicrobial agents can cause a significant reduction in the amount of ergosterol, a major sterol component responsible for maintaining cell function and integrity in the fungal cell membrane [40]. Most of the previous studies on rosemary essential oil focused on the antibacterial role. Sirocchi *et al*. [41] illustrated that incorporation of *R. officinalis* at only 4% (w/w) to active packaging preserved meats from damage by the cadaverine-producer bacteria *Brocothrix thermospacta* or any of Enterobacteriaceae members. Sachets that provide a slow discharge of *R. officinalis* essential oil can also be combined into packaged meat products and used to prevent the meats' putrefaction [42]. The culinary and medicinal uses of rosemary herb are due to its massive arrays of chemical components known as plant metabolites. Among these arrays are low- molecular-weight aromatic chemical compounds called essential oils, and these play a fundamental role in the culinary and antimicrobial properties of the plant [13]. Rosemary essential oil is dominated by α-pinene, 1,8- cineole, α- terpineol, camphene, and borneol as main compounds [43], and these bioactive compounds are responsible for the different medicinal effects of the general antimicrobial [15-17] and antioxidant [14] activities, in addition to other activities, *e.g*. anticarcinogenic properties [44]. Polyphenolic compounds are another group of components found in rosemary essential oil; however, in recent years the most important of rosemary constituents that have gained significant attention is the unique class of terpenes [45]. Our data revealed that eucalyptol is one of the dominant constituents (11.21%) in rosemary oil (Table 2). It has been well known that terpenes possess antifungal properties. Interestingly, some fungal species were recently discovered to produce eucalyptol in large amounts [46]. In the current detection, thirty-two constituents were identified. Only four constituents, pinene, camphene, limonene, and eucalyptol represent more than 50% of all the chemical composition in rosemary essential oil. Camphore type was the most frequent skeletal sesquiterpene in rosemary oil composition. Fig. 4 displays the main sesquiterpene skeletons of rosemary oil. The chemical analysis of rosemary oil is shown in Table 2. Fu *et al*. [47] estimated that the main compositions in rosemary oil were cineole (27.2%), pinene (19.4%), camphor (14.3%), camphene (11.5%), Borneol (3.2%), β-Caryophyllene (2.41%), and Bornyl acetate (1.13%). Sienkiewicz *et al*. [48] reported that the essential oil of rosemary contains thirty-seven constituents with the main ones being cineole (46.5%), camphor (11.5%), pinene (11.1%), β-pinene (9.1%) and camphene (5.3%). The differences in chemical compositions of rosemary oil may be partially due to the variation in the preparation technique, geographical source and maturity stage of *R. officinalis* plant. Nevertheless, the crude essential oil displayed growth inhibition influence on all pathogenic fungal species examined in the present study (Fig. 5). This study also suggests good examples of antifungal substances from rosemary and *Trichoderma* which are biodegradable natural products that may be safe for application soon. In conclusion, the fungicidal effects of *R. officinalis* L. essential oil, the VOCs of *T. koningiopsis* T-403, and the fungicides mancozeb and fenhexamid, were evaluated to suppress the severe fungal pathogens causing the ginseng root rot. Rosemary oil was effective to prevent the growth of phytopathogenic fungi. *S. nivalis* exhibited the lowest MIC. GC-MS analysis showed the presence of thirty-two constituents within the essential oil of *R. officinals*, and some of these are documented to possess antimicrobial properties, e.g. eucalyptol. Our results may assert the importance of essential oils found in common plants in the protection of ginseng crops from mycopathogens. This study will help in the prospective development of biopesticides for the eco-friendly control of plant pathogens and proposes the potential use of the antimicrobial compound from *Trichoderma*.

## Figures and Tables

**Table 1:** Antifungal indices of rosemary essential oil.

Fungal pathogens	Rosemary oil concentration (%)				*T.koningiopsis* T-403	Mancozeb	Fenhexamid

	0.05	0.1	0.3	0.5	VOCs	20 μgml^-1^	20 μgml^-1^
*Botrytis cinerea*	5.7 ± 0.21e	17.5 ± 0.06f	23.3 ± 0.26d	52.2 ± 0.59c	54.4 ± 2.48ab	1.0 ± 0.58e	0.0 ± 0.00b
*Alternaria panax*	49.7 ± 0.59a	91.5 ± 0.15b	100 ± 0.00a	100 ± 0.00a	67.5 ± 3.72a	45.0 ± 2.00c	30.0 ± 1.00d
*Cylindrocarpon destructans*	33.1 ± 0.07b	45.8 ± 0.80c	49.7 ± 0.25b	66.4 ± 0.32b	84.0 ± 22.63a	35.0 ± 0.58d	20.0 ± 2.00d
*Fusarium oxysporum*	6.1 ± 0.15e	18.7 ± 0.21e	24.9 ± 0.15c	43.4 ± 0.40d	63.9 ± 1.96b	10.0 ± 1.00d	13.0 ± 0.58ab
*Sclerotinia sclerotiorum*	17.4 ± 0.32c	23.5 ± 0.10d	100 ± 0.00a	100 ± 0.00a	54.7 ± 30.78ab	19.0 ± 0.58d	13.0 ± 2.00d
*Sclerotinia nivalis*	16.5 ± 0.06d	100 ± 0.00a	100 ± 0.00a	100 ± 0.00a	57.1 ± 14.14ab	1.0 ± 0.58c	0.0 ± 0.00d

Means with the same letter within a column are not significantly different at *p* < 0.05.

**Table 2:** Chemical compositions of rosemary essential oil.

Peak No.	Compound	RT (min)	Concentration (%)
1	Tricyclo[2.2.1.0(2,6)]heptane,1,3,3-trimethyl-	02:18	0.14
2	Spiro[4.5]dec-1-ene	02:46	0.1
3	Tricyclo[2.2.1.0(2,6)]heptane, 1,7,7-trimethyl-	03:02	5.14
4	a-Pinene	03:14	12.8
5	Bicyclo[2.2.1]heptane,7,7-dimethyl-2-methylene-	03:31	1.26
6	Camphene	03:53	11.04
7	b-Pinene	04:31	8.09
8	Bicyclo[3.1.0]hexane, 4-methylene-1-(1-methylethyl)-	04:49	2.03
9	b-Phellandrene	04:56	0.08
10	a-Pinene	05:23	2
11	a-Phellandrene	05:44	1.01
12	D-Limonene	06:30	6.16
13	Bicyclo[3.1.0]hex-2-ene, 4-methyl-1-(1-methylethyl)-	06:43	0.08
14	Eucalyptol	06:59	11.21
15	1,4-Cyclohexadiene, 1-methyl-4-(1-methylethyl)-	07:36	0.72
16	Benzene, 1-methyl-3-(1-methylethyl)-	08:15	7.48
17	Cyclohexene, 4-methyl-3-(1-methylethylidene)-	08:30	0.32
18	Cyclohexene, 1-methyl-4-(1-methylethylidene)-	08:38	0.04
19	3-Oxatricyclo[4.1.1.0(2,4)]octane,2,7,7-trimethyl-O	11:01	0.07
20	Bicyclo[2.2.1]heptan-2-one, 1,3,3-trimethyl-	11:40	0.08
21	Acetaldehyde, (3,3-dimethylcyclohexylidene)-, (E)-	12:56	0.71
22	Bicyclo[2.2.1]heptan-2-one, 1,7,7-trimethyl-, (1R)-	14:48	12.33
23	1,6-Octadien-3-ol, 3,7-dimethyl-	15:24	1.1
24	1,6-Octadien-3-ol, 3,7-dimethyl-, 2-aminobenzoate	15:34	0.31
25	Aceticacid,1,7,7-trimethyl-bicyclo[2.2.1]hept-2-ylester	16:16	4.14
26	3-Cyclohexen-1-ol, 4-methyl-1-(1-methylethyl)-	16:46	0.12
27	Bicyclo[2.2.1]heptan-2-one, 1,7,7-trimethyl-, (1S)-	17:10	0.26
28	Cyclohexanol, 1-methyl-4-(1-methylethenyl)-	17:27	0.15
29	a-Caryophyllene	17:58	0.38
30	Isoborneol	18:05	0.6
31	cis-b-Terpineol	18:43	0.12
32	3-Cyclohexene-1-methanol, a,a4-trimethyl-	19:02	9.96
